# Solution Structure of Nucleoprotein Domain 1 from the Emerging Yezo Virus

**DOI:** 10.3390/ijms27125492

**Published:** 2026-06-18

**Authors:** Anastasia V. Gladysheva, Alexey O. Yanshin, Nikita S. Radchenko, Irina A. Osinkina, Egor O. Ukladov, Alexander P. Agafonov

**Affiliations:** 1 State Research Center of Virology and Biotechnology “Vector”, 630559 Kol’tsovo, Russia; yanshin_ao@vector.nsc.ru (A.O.Y.); radchenko_ns@vector.nsc.ru (N.S.R.); osinkina_ia@vector.nsc.ru (I.A.O.); agafonov@vector.nsc.ru (A.P.A.); 2 Physics Department, Novosibirsk State University, 630090 Novosibirsk, Russia; 3 Scientific Educational Center of the Institute of Chemical Technology, Novosibirsk State University, 630090 Novosibirsk, Russia; e.ukladov@g.nsu.ru; 4 Siberian Ring Photon Source “SKIF”, 630559 Kol’tsovo, Russia

**Keywords:** *Ixodid tick*, tick-borne virus, RNA virus, *Nairoviridae*, *Orthonairovirus*, viral protein, ribonucleoprotein complex, protein structure, small-angle X-ray scattering

## Abstract

The Yezo virus (YEZV) is a recently discovered tick-borne orthonairovirus with pathogenic potential, causing acute febrile illness in humans. Viral nucleoproteins (N) play a key role in genome packaging, replication, and modulation of host immune responses, making their structural characterization essential for understanding viral pathogenesis and developing targeted countermeasures. However, the absence of structural data for YEZV proteins significantly hinders these efforts. This study presents the first solution structure of the YEZV N domain 1 (D1). A highly purified, soluble, tag-free recombinant YEZV N D1 was produced from the native sequence of the clinical YEZV isolate. The native-state conformation was resolved through an integrated approach combining size-exclusion chromatography coupled with small-angle X-ray scattering (SEC-SAXS), AlphaFold 3 structure prediction, and all-atom molecular dynamics simulations. The YEZV N D1 structure adopts a stable, predominantly α-helical globular fold that remains monomeric under near-physiological conditions. SEC-SAXS data show excellent agreement with computational models, revealing moderate conformational flexibility. The characterized recombinant YEZV N D1 and its first solution structure reported here providing essential insights into understanding of YEZV molecular architecture. These findings lay a foundation for rational serological assay development and structure-guided therapeutic design against this and other emerging orthonairoviruses.

## 1. Introduction

The Yezo virus (YEZV; *Orthonairovirus yezoense*, *Orthonairovirus* genus, *Nairoviridae* family) is an emerging tick-borne pathogen first identified in humans in Hokkaido, Japan (2021) [1]. Infection typically presents as an acute febrile illness characterized by high fever, thrombocytopenia, leukopenia, elevated hepatic transaminases, and marked hyperferritinemia, with a median incubation period of ~7 days following tick exposure [1]. In contrast to several highly pathogenic orthonairoviruses, reported YEZV infections have not exhibited hemorrhagic manifestations, and all documented patients have achieved complete clinical recovery within 2–3 weeks [1,2]. Nevertheless, the expanding geographic footprint and zoonotic potential of YEZV have positioned it as a notable emerging public health concern.

Since its initial discovery, YEZV has been documented across multiple countries, ecological niches, and host species. In Japan, 22 confirmed human cases have been reported, predominantly in Hokkaido, with disease onset tightly correlated with the seasonal activity of *Ixodes* ticks [1]. In northeastern China, at least 22 human cases have been identified, including retrospective infections from 2012. Clinical manifestations in Chinese patients are generally milder than those observed in Japan [1,2]. In Russia, YEZV was first isolated in 2024 from *Ixodes persulcatus* ticks, representing the westernmost known distribution limit at the time of reporting [3]. Subsequent metagenomic surveillance identified a novel YEZV genetic variant in *Ixodes pavlovskyi* ticks in the Tomsk region (Russia), expanding geographic distribution [2]. Beyond human and tick hosts, YEZV RNA or specific antibodies have been detected in wild mammals, including sika deer (*Cervus nippon yesoensis*), raccoons (*Procyon lotor*), and raccoon dogs (*Nyctereutes procyonoides*) [3]. Additionally, viral genomes have been identified in ticks collected from migratory passerines, such as black-faced buntings (*Emberiza spodocephala*), supporting a role for avian migration in the cross-regional dispersal of YEZV [1,3]. Recent metagenomic analysis of a gray seal from the Baltic Sea led to the proposal of a novel Östhammar virus (*Orthonairovirus östhammarense*), which shares ~90% identity with YEZV and phylogenetically clusters within the same Eurasian clade of human-pathogenic orthonairoviruses [4]. This discovery demonstrates that the viral lineage can infect a wide range of vertebrate hosts, including marine mammals. It also emphasizes the urgent need to characterize conserved structural elements. Understanding these features will help elucidate the mechanisms of cross-species adaptation. Ultimately, such knowledge will directly inform the development of broad-spectrum antiviral strategies.

The YEZV genome comprises three negative-sense, single-stranded RNA (ssRNA(-)) segments: small (S), medium (M), and large (L) [3]. The S segment (~1.6 kb) encodes the nucleoprotein (N). The M segment (~4.0 kb) encodes the glycoprotein precursor (GPC), which is post-translationally processed into surface glycoproteins Gn and Gc. The L segment (~12.0 kb) encodes a multifunctional L protein containing an RNA-dependent RNA polymerase (RdRp) and an OTU-like protease domain implicated in suppressing host innate immune responses [2,3]. The 3′ and 5′ termini of each segment share partial complementarity, enabling intramolecular base-pairing to form a conserved panhandle promoter structure essential for viral transcription and genome replication [3,5].

The N is the most abundant viral protein and serves as the foundational structural component of the viral ribonucleoprotein (RNP) complex. By encapsidating the viral genomic RNA, N shields it from degradation by cellular nucleases and provides the essential structural template for the viral RdRp to initiate transcription and replication [5,6]. Structural studies of related orthonairoviruses reveal that RNPs adopt flexible, ring-like oligomeric architectures. This conformational plasticity likely facilitates dynamic interactions with the viral polymerase and host cellular machinery [5,7].

Structural analyses of related virus N, including those from Crimean-Congo hemorrhagic fever virus (*Orthonairovirus haemorrhagiae*; CCHFV), Songling virus (*Orthonairovirus songlingense*; SGLV), and Beiji nairovirus (*Norwavirus beijiense*; BJNV), demonstrate a highly conserved “racket-shaped” tertiary architecture composed of a compact globular “head” domain (Domain 2, D2) and an elongated “stalk” domain (Domain 1, D1) [3,5,7]. The N D1 protrudes from the globular body and mediates critical head-to-tail intermolecular contacts that drive oligomerization into the superhelical RNP [5,6]. In CCHFV, this region contains a conserved caspase-3 cleavage site characterized by a DEVD motif. In the oligomeric state, this site remains sterically occluded. However, the binding of primer-length RNA triggers a conformational switch that exposes the motif. This structural rearrangement directly links RNP dynamics to host antiviral defense mechanisms [5]. Similarly, integrative structural studies of SGLV N and BJNV N confirm that the D1 exhibits pronounced conformational flexibility, adopting distinct orientations relative to the D2 that likely regulate RNA-binding affinity and monomer–oligomer transitions [7]. Positively charged residues within the D1 contribute to a continuous RNA-binding crevice that runs along the interior of the RNP, shielding the viral genome and facilitating specific recognition of the terminal panhandle structure [6,7]. Notably, immunoinformatic predictions for YEZV N identify several candidate B- and T-cell epitopes within the YEZV N D1 [8,9]. Therefore, elucidating the structural dynamics of the corresponding domain in the emerging YEZV is critical for understanding its pathogenesis and identifying conserved targets for broad-spectrum antiviral interventions.

These observations strongly justify a detailed biophysical characterization of the YEZV N D1. To elucidate the structural features of the YEZV N D1, we applied an integrated approach combining small angle X-ray scattering with size exclusion chromatography (SEC-SAXS), tertiary structure prediction using AlphaFold 3, and molecular dynamic (MD) simulations. The recombinant YEZV N D1 and its first solution structure reported here provide a foundation for understanding the molecular architecture of YEZV, development of serological assays and structure-guided therapeutic design against this and other emerging orthonairoviruses.

## 2. Results

### 2.1. Characteristics of the Recombinant YEZV Nucleoprotein Domain 1

The recombinant YEZV N D1 was produced using the native (non-codon-optimized) nucleotide sequence corresponding to the YEZV HH009-2017 isolate, originally identified in a patient in Japan in 2017 [10].

**Figure 1:**
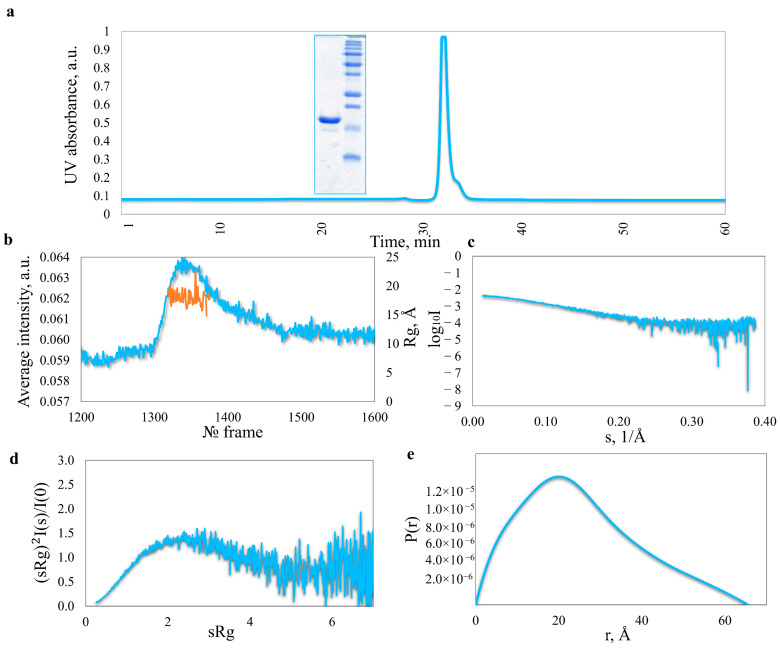
Size-exclusion chromatography (SEC) coupled with small-angle X-ray scattering (SAXS) analysis of Yezo virus (YEZV) nucleoprotein (N) domain 1 (D1). (**a**) Ultraviolet (UV) SEC chromatogram and sodium dodecyl sulfate-polyacrylamide gel electrophoresis (SDS-PAGE) of recombinant YEZV N D1. (**b**) SEC-SAXS chromatogram of YEZV N D1. The orange line indicates Rg. (**c**) Experimental SAXS profile of YEZV N D1. (**d**) Dimensionless Kratky plot of YEZV N D1. (**e**) Pair distance distribution function, P(r), of YEZV N D1.

We successfully obtained highly purified (>95% by SDS-PAGE) recombinant YEZV N D1 (125 aa, pI 7.91) in a soluble, tag-free form using a prokaryotic expression system. SEC on a Superdex^®^200 Increase 10/300 GL column yielded a single symmetric elution peak at 18.1 mL, consistent with the expected monomeric molecular weight of 14.3 kDa (Figure 1a). The A260/A280 absorbance ratio of ~0.5 indicates that the YEZV N D1 was successfully stripped of endogenous nucleic acids from the expression host. Both SEC and DLS analysis demonstrated that YEZV N D1 predominantly exist in a monomeric state in solution, exhibiting a hydrodynamic radius (Rh) of 18.6 ± 4 Å (Figure S1).

### 2.2. SEC-SAXS Study of the Recombinant YEZV Nucleoprotein Domain 1 in Solution

To establish a structural baseline for the YEZV N, we first determined the experimental solution-state conformation of its recombinant D1. Due to the YEZV N D1 propensity for aggregation, SEC-SAXS was specifically employed to isolate the monodisperse fraction for scattering measurements.

A total of 2260 SAXS frames were collected continuously during sample elution. Buffer frames were selected from regions both before (1150–1250 frames) and after (1550–1650 frames) the YEZV N D1 elution peak to account for potential baseline drift during the SEC-SAXS. Sample frames were selected from the peak apex (1300–1380 frames), where Rg was stable. The same scattering curves were obtained when using only the pre-peak buffer region (1150–1250 frames) for subtraction, confirming that baseline drift was negligible and that the selected buffer region accurately represented the background (Figure 1b,c).

Chromatographic profiling revealed a single, symmetric elution peak, indicating sample homogeneity (Figure 1b). The Kratky plot displayed a slight shift in the peak maximum relative to the theoretical profile for an ideal compact globule, suggesting the presence of flexible loop regions (Figure 1d). The P(r) exhibited a single, well-defined maximum, consistent with a monodisperse, predominantly globular particle (Figure 1e).

Quantitative analysis of the SAXS data yielded Rg = 19 ± 1 Å and Dmax = 65 Å for the experimental ensemble. These values are in excellent agreement with the corresponding parameters calculated from the AlphaFold-predicted YEZV N D1 model (Rg = 18 ± 1 Å, Dmax = 65 Å), supporting the conclusion that the recombinant YEZV N D1 adopts in solution a conformation closely resembling the computationally predicted fold. The SAXS-derived Rg is also consistent with the Rh obtained by DLS, yielding an Rg/Rh ratio of ~1.0, which is characteristic of a compact, predominantly globular particle with limited conformational flexibility. The MW estimated from the SAXS data (13.8 kDa, CI: 13.1–14.8) is consistent with the theoretical MW = 14.3 kDa.

**Figure 2:**
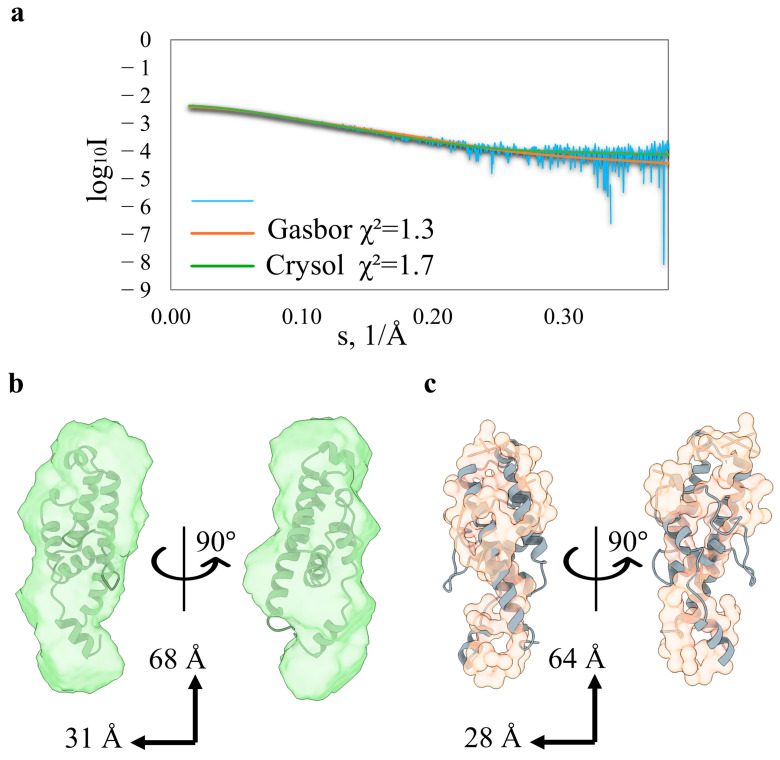
(**a**). Calculated theoretical scattering profiles from ab initio structures modeled using GASBOR (orange) and CRYSOL (green), superimposed on the experimental SAXS profile of YEZV N D1 (blue). (**b**). Low-resolution ab initio shape of YEZV N D1 in solution, reconstructed using DAMMIN and dummy atom modeling (green surface). (**c**). Low-resolution ab initio shape of YEZV N D1 in solution, reconstructed using GASBOR and dummy residue model (orange surface). The AlphaFold-predicted YEZV N D1 structure is displayed as ribbon diagrams.

The experimental scattering curves in the measured s-range showed good agreement with theoretical profile calculated from the AlphaFold-predicted YEZV N D1 structure. The computational YEZV N D1 model exhibited a high predicted Local Distance Difference Test (pLDDT = 88) and predicted Template Modeling score (pTM = 0.81), indicating a reliable global fold prediction. Inspection of residue-level confidence scores revealed that the α-helical core was predicted with uniformly high confidence, whereas lower confidence values were primarily localized to the N- and C-terminal regions. Validation using CRYSOL yielded a χ^2^ value of 1.7, while GASBOR resulted in an improvement in the χ^2^ value to 1.3 (Figure 2a).

Ab initio low-resolution shape reconstruction was performed by approximating the molecular envelope using both dummy-atom and dummy-residue modeling approaches. A total of 15 independent reconstructions were generated to identify the most stable and reproducible conformation. The resulting low-resolution envelope (~25 Å) in solution exhibits an elongated overall shape, consistent with the predicted compact α-helical globular tertiary structure of YEZV N D1. Notably, the dummy-residue-derived YEZV N D1 envelope yielded a more compact molecular volume compared to the dummy-atom model, a difference likely attributable to the higher spatial granularity inherent to residue-level bead representation (Figure 2b,c).

### 2.3. MD Simulations of the YEZV Nucleoprotein Domain 1 Structure

**Figure 3:**
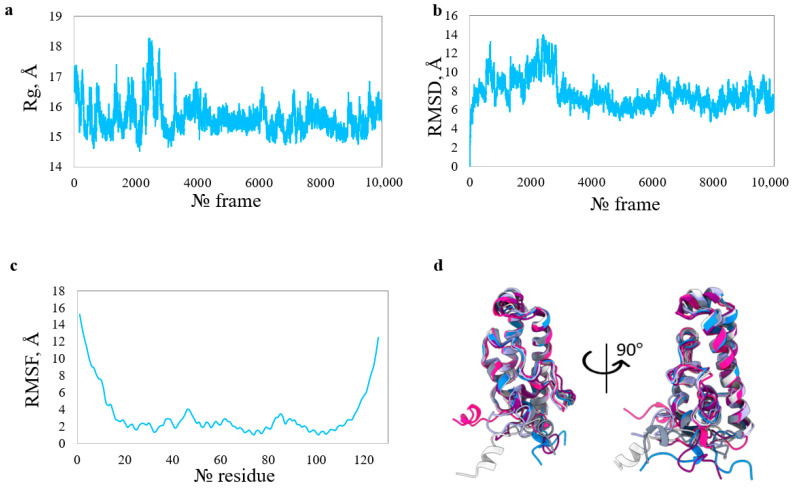
Conformational dynamics of YEZV N D1 revealed by 500 ns MD simulations. (**a**) Time evolution of the Rg. (**b**) RMSD of Cα atoms relative to the initial structure, plotted against trajectory frame number. (**c**) RMSF profile for amino acid residues. (**d**) Structural superposition of representative trajectory frames shown in two orthogonal projections (rotated by 90°), with each structure colored differently.

All-atom MD simulations of YEZV N D1 were performed for 500 ns to assess conformational stability in solution. The structure remained stable throughout the trajectory, with root-mean-square deviation (RMSD) values reaching a plateau in the range of approximately 3.5–5.5 Å following an initial relaxation phase (Figure 3a). The Rg fluctuated within a relatively narrow range of 15.0–18.5 Å. Minor fluctuations in Rg were attributed to dynamic motions of surface loops and terminal regions (Figure 3b). Root-mean-square fluctuation (RMSF) analysis revealed that the majority of secondary structure elements exhibited low mobility (1–2 Å). RMSF values were predominantly localized to the N- and C-terminal segments and selected loop regions, where fluctuations reached 10.0–17.0 Å (Figure 3c).

Superposition of YEZV N D1 trajectory frames confirmed high conservation of the overall domain architecture (Figure 3d). The α-helices retained their relative positions. Structural differences were driven primarily by flexibility in the terminal segments and inter-helical loops.

The Rg plot indicates that the YEZV N D1 does not reach equilibrium within the first 150 ns. Therefore, the trajectory segment from 150 to 500 ns was used for further analysis.

### 2.4. Scattering Profiles from MD Simulations of the YEZV Nucleoprotein Domain 1 Structure

**Figure 4:**
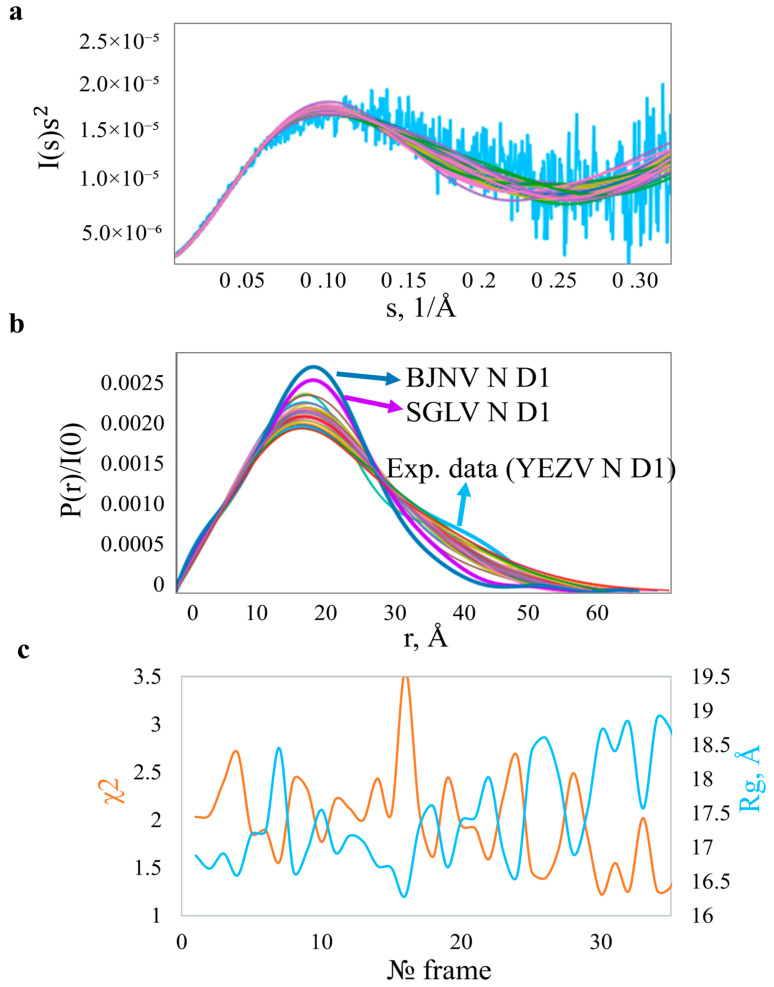
Comparison of experimental SEC-SAXS data for YEZV N D1 with theoretical scattering profiles derived from MD trajectories. (**a**) Normalized scattering curve in Kratky coordinates showing the experimental scattering curve (blue line) overlaid with theoretical profiles calculated for representative MD frames (colored lines). (**b**) P(r) for the experimental YEZV N D1 data (blue line), selected YEZV N D1 trajectory frames (colored lines), and AlphaFold3-derived theoretical profiles for BJNV N D1 (dark blue line) and SGLV N D1 (purple line). (**c**) Dynamics of the χ^2^ (orange line) and Rg (blue line) across the analyzed MD trajectory frames.

To sample the conformational states populated during the 500 ns MD simulation of YEZV N D1 structure, theoretical SAXS scattering profiles and P(r) were calculated for representative trajectory frames and subsequently compared with SEC-SAXS data (Figure 4a). Similar calculations were performed for homologous viral proteins of BJNV N D1 and SGLV N D1, enabling inter-protein comparison of geometric parameters and folding features [7].

Analysis of the P(r) functions revealed that YEZV N D1, BJNV N D1, and SGLV N D1 share a similar overall organization but differ quantitatively in their geometric parameters. For YEZV N D1, the experimental SAXS-derived P(r) exhibits a slightly broader distribution with an extended tail at higher distances, consistent with a more elongated overall shape (Figure 4b). In contrast, the P(r) profiles for BJNV N D1 and SGLV N D1 are more symmetric and correspond to more compact globular architectures. BJNV N D1 displayed Rg = 17 Å and Dmax = 64 Å, whereas SGLV N D1 showed Rg = 16 Å and Dmax = 60 Å. Thus, the Rg of YEZV N D1 is approximately 11% larger than that of BJNV N D1 and 17% larger than that of SGLV N D1. Similarly, the Dmax of YEZV N D1 exceeds that of BJNV N D1 by ~2% and that of SGLV N D1 by ~8%. These quantitative differences are consistent with the broader P(r) distribution and extended high-distance tail observed for YEZV N D1, supporting a slightly more elongated molecular architecture relative to the homologous domains.

The χ^2^ between YEZV N D1 theoretical SAXS scattering profiles computed from MD frames and the experimental SAXS profiles varied across the trajectory in the range of ~1.25–3.58 (Figure 4c). Minimal χ^2^ values were achieved for conformations with Rg = 18.0–19.0 Å, indicating the existence of a preferred compactness range that best agrees with the experimental solution-state data.

### 2.5. YEZV Nucleoprotein Domain 1 RNA-Binding Analysis

**Figure 5:**
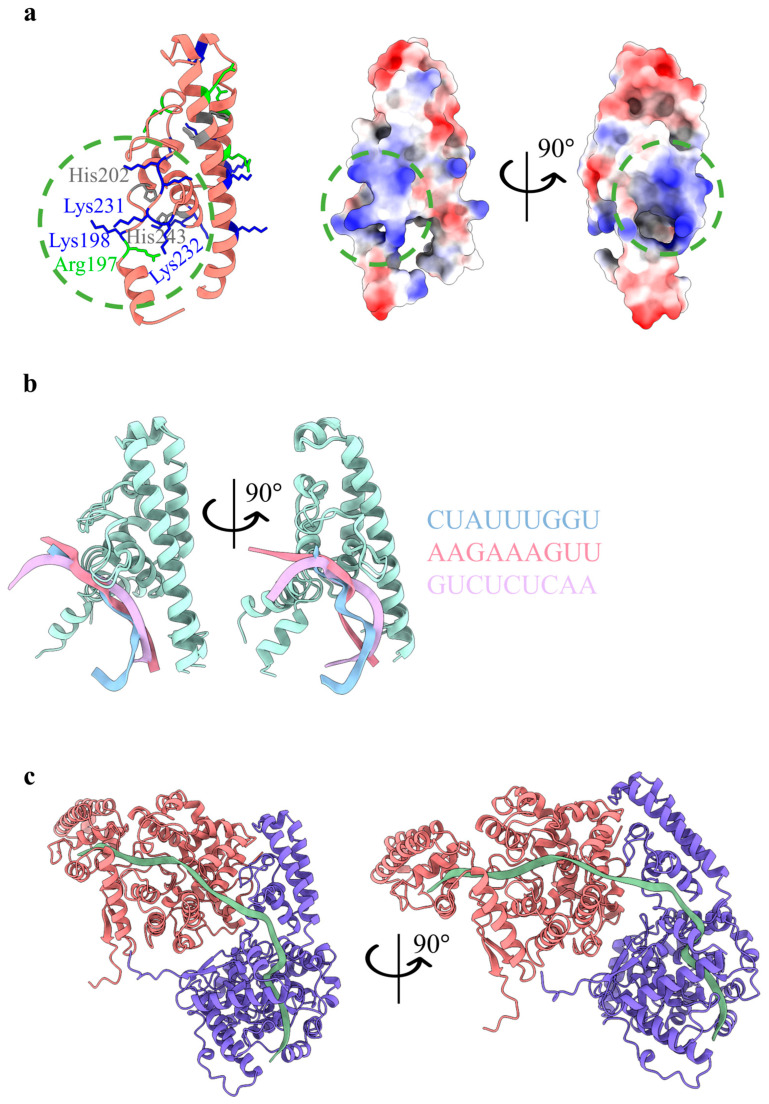
Analysis of RNA-binding by YEZV N. (**a**) Structural model of the YEZV N D1. Left: ribbon representation of the predicted YEZV N structure. Conserved residues are shown as sticks and labeled. Right: electrostatic surface representation of the same region shown in two orientations rotated by 90°. Blue and red surfaces indicate positively and negatively charged regions, respectively. The putative RNA-binding site highlighted in a green dashed circle. (**b**) Structural model of the YEZV N D1 (light green) with different ssRNA(-). The YEZV N D1 is shown in cartoon representation. RNA molecules are colored individually. (**c**) Structural model of the YEZV N dimer in complex with ssRNA(-). YEZV N monomers are shown in different colors (pink and purple), and the RNA is indicated in green.

To evaluate the RNA-binding capacity of YEZV N D1, surface electrostatic potential analysis was performed, followed by modeling of complexes between YEZV N D1 and fragments of its genomic ssRNA(-). YEZV N D1 features a distinct positively charged surface patch formed by conserved residues Arg197, Lys198, His202, Lys231, and Lys232 (Figure 5a). Structural modeling of YEZV N D1 in complex with three distinct ssRNA(-) confirmed specific protein–RNA interactions localized to this cationic region (Figure 5b and Figure S2). The predicted monomeric complex was generated with high confidence metrics (ipTM = 0.77, pTM = 0.83). Structural alignment further revealed that the RNA-binding interface of YEZV N D1 is spatially conserved and corresponds to the functionally equivalent regions in the CCHFV N D1 and Hazara virus (*Orthonairovirus hazaraense*) N D1. Modeling of a YEZV N dimer in complex with ssRNA(-) (ipTM = 0.75, pTM = 0.81) demonstrated that the same positively charged patch remains fully accessible and effectively mediates RNA-binding in the oligomeric state (Figure 5c and Figure S3).

**Figure 6:**
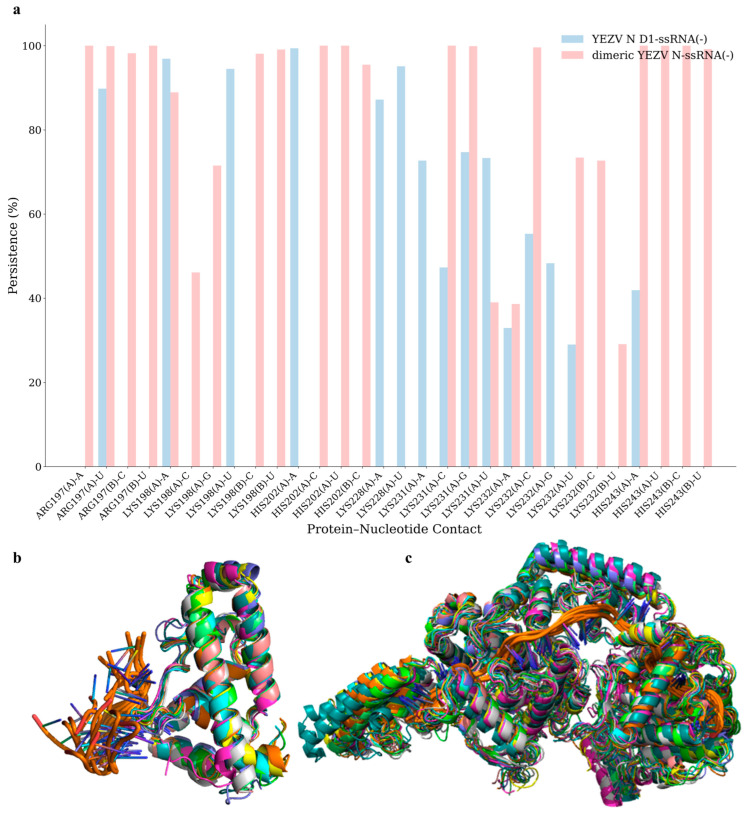
MD simulations of the YEZV N D1-ssRNA(-) complex and the dimeric YEZV N-ssRNA(-) complex. (**a**) Comparison of persistent protein–RNA interactions involving Arg197, Lys198, His202, Lys228, Lys231, Lys232, and His243 in two molecular dynamics conditions: YEZV N D1-ssRNA(-) (blue bars) and dimeric YEZV N-ssRNA(-) (pink bars). Only protein–nucleotide contacts with persistence >20% were included. (**b**) Structural superposition of representative conformations of the YEZV N D1-ssRNA(-) complex obtained from RMSD-based clustering. (**c**) Structural superposition of representative conformations of the dimeric YEZV N-ssRNA(-) complex obtained from RMSD-based clustering, with each structure colored differently.

MD analysis revealed that the YEZV N D1-ssRNA(-) complex is stabilized by a defined group of persistent protein–nucleotide contacts (Figure 6a,b, and Figure S4). In this system, the most stable interactions were formed by Arg197, Lys198, His202, and Lys228. His202-A contacts reached 98.2% persistence, while Lys198 formed highly persistent interactions with A and U, including Lys198-A contacts up to 96.9% and Lys198-U contacts up to 94.5%. Lys228 also contributed to RNA stabilization, forming persistent contacts with U and A, including Lys228-U contacts up to 95.1% and Lys228-A contacts up to 87.2%. Arg197 was also involved in RNA through contacts with U (89.8%). Lys231 formed moderately to highly persistent contacts with G, U, A, and C, with the strongest values around 72.7–74.7%, whereas Lys232 showed lower-to-moderate persistence. His243 contributed only a moderate contact.

In the dimeric YEZV N-ssRNA(-) MD simulation, the protein–RNA interaction network became more extensive (Figure 6a,c, and Figure S5). The most prominent change was the strong involvement of Arg197, which formed highly persistent contacts in both chains. Arg197 contacts with U, A, and C reached 97.2–100.0%, indicating that Arg197 is a major RNA-anchoring residue in the dimeric RNP. His202 also remained an important RNA-binding residue, forming highly persistent contacts with C and U, including His202-C and His202-U contacts reaching 95.5–100.0%. In contrast to the YEZV N D1-ssRNA(-) complex, His243 became a major contributor in the dimeric YEZV N-ssRNA(-) state, forming near-complete contacts with A, C, and U in both chains. Lys231 was also strongly stabilized in the dimeric RNP complex, especially through Lys231-C and Lys231-G contacts, which reached 99.8–100.0%. Lys232 formed persistent contacts mainly with C and U, including Lys232-C at 99.6% and Lys232-U at 73.4%. Notably, Lys228, which was one of the dominant residues in the monomeric YEZV N D1-ssRNA(-) complex, was not detected among persistent dimeric contacts above the 20% threshold, suggesting that RNA engagement is redistributed upon YEZV RNP oligomerization.

## 3. Discussion

Orthonairovirus N proteins represent one of the most functionally critical and structurally conserved components across the *Nairoviridae* family, serving as essential mediators of viral genome packaging, replication, and host immune modulation [11]. These proteins play central roles in the viral life cycle, from initial RNA encapsidation to the formation of functional RNP complexes that serve as templates for viral transcription and replication [12,13]. The N protein’s multifunctional nature extends beyond RNA binding to include modulation of host cellular processes, immune evasion mechanisms, and facilitation of viral assembly and release [14,15]. Within this broader context of orthonairovirus nucleoprotein biology, the structural characterization of YEZV N assumes particular significance. YEZV represents an emerging tick-borne orthonairovirus that has recently expanded its geographical range from its initial discovery in Japan to include detection across China and, most recently, Russia [2,3]. This geographic expansion, coupled with the virus’s association with acute febrile illness in humans, positions YEZV as a pathogen of growing public health concern.

This study presents the first experimental structural characterization of the YEZV N D1, providing crucial insights into the molecular architecture of this emerging tick-borne pathogen. Orthonairovirus nucleoproteins are highly immunogenic and serve as major targets for both humoral and cellular immune responses [16,17]. The CCHFV N D1 was shown to be essential for Th17 cell activation, particularly through promoting IL-17A production [18]. The high immunogenicity of orthonairovirus N D1 and their role as dominant antigens in natural infections make them ideal targets for both IgM and IgG detection assays. A distinctive feature of this study is the use of the native, non-codon-optimized nucleotide sequence encoding YEZV N D1 derived from the HH009-2017 isolate, originally identified in a patient in Hokkaido, Japan. Biophysical data obtained for the native YEZV N D1 sequence ensure direct relevance to the naturally circulating YEZV. This is particularly important for the development of ELISA diagnostic assays, where antibodies raised against a consensus variant might exhibit reduced affinity for the wild-type antigen. However, potential cross-reactivity with other orthonairovirus N must be carefully considered in assay design, particularly in regions where multiple orthonairoviruses co-circulate [19].

Our structural characterization of YEZV N D1 demonstrates remarkable agreement between experimental SEC-SAXS data and AlphaFold 3 predictions, with χ^2^ value of 1.3. This level of agreement is particularly noteworthy when compared to similar studies of related orthonairovirus nucleoproteins. The modest improvement in fit quality for GASBOR (χ^2^ = 1.3) relative to CRYSOL (χ^2^ = 1.7) reflects methodological differences. CRYSOL computes scattering from a single static atomic model, whereas GASBOR reconstructs a low-resolution envelope directly optimized against experimental data, implicitly accounting for solution-averaged conformational heterogeneity. This interpretation is consistent with independent evidence from Kratky analysis and MD simulations, which indicate limited flexibility in surface loops and terminal regions of YEZV N D1.

The high pLDDT score and close correspondence of key structural parameters validate the computational model and demonstrate the reliability of current AI-based structure prediction methods for proteins of novel viruses. These results can be directly compared with our recent structural characterization of SGLV N and BJNV N, which employed identical SAXS and computational modeling approaches [7]. The consistency of experimental–computational agreement across these three emerging members of *Nairoviridae* family validates the methodology and establishes a robust framework for rapid structural characterization of recently discovered viruses.

The MD simulation results reveal important insights into YEZV N D1 dynamics. The structural stability observed over 500 ns indicates a well-folded, thermodynamically stable domain capable of maintaining its functional conformation under physiological conditions. The localized flexibility in terminal regions and inter-helical loops suggests these regions may serve as hinge points for conformational changes during RNA-binding or protein–protein interactions.

The comparison with homologous domains from BJNV and SGLV reveals interesting evolutionary and functional relationships within the *Nairoviridae* family. While all three domains share similar overall organization and dimensions, the distinct P(r) profiles indicate subtle but potentially important structural differences. The more elongated shape of YEZV N D1 compared to the more compact architectures of BJNV and SGLV domains may reflect adaptation to specific RNA-binding requirements. However, it is important to note a key limitation. The theoretical scattering curves profiles for BJNV N D1 and SGLV N D1 were derived exclusively from computational structural models rather than experimental SAXS data. Consequently, these predictions may not fully reflect the actual BJNV N D1 and SGLV N D1 solution-state structures.

The identification of a conserved cationic patch in YEZV N D1 aligns with our SEC-SAXS and MD observations of moderate flexibility in surface loops, suggesting that dynamic rearrangements may facilitate RNA capture and release during RNP assembly. Given the conservation of this RNA-binding interface across pathogenic orthonairoviruses, the characterized cationic patch represents a promising target for structure-guided development of antivirals [20]. While computational modeling provides high-confidence predictions, experimental validation of RNA-binding affinity (electrophoretic mobility shift assays, surface plasmon resonance, or isothermal titration calorimetry) and mapping of contact residues by mutagenesis will be essential in future. Rigorous experimental validation will therefore be essential to confirm the biological relevance of this RNA-binding interface.

As genomic surveillance continues to uncover novel tick-borne orthonairoviruses, the methodological and structural framework established here will accelerate the characterization of emerging viral proteins and support the rapid development of targeted countermeasures.

## 4. Materials and Methods

### 4.1. Construction of the YEZV Nucleoprotein Domain 1 Expression Plasmid

A DNA copy of the sequence encoding YEZV N D1 (GenBank ID: LC628645) was synthesized de novo by assembly from pairwise overlapping oligonucleotides followed by PCR amplification (375 bp in length) with Q5 High-Fidelity DNA Polymerase (NEB, Hitchin, UK). The resulting DNA fragment was directionally cloned into the pJET1.2/blunt (Invitrogen, Carlsbad, CA, USA) for sequence verification and long-term storage. Subsequently, it was PCR-amplified to introduce a cleavage site for the rhinovirus A28 3C protease and a CACC overhang for D-TOPO cloning. The target fragment was directionally ligated into the pET200 D-TOPO vector (Invitrogen, Carlsbad, CA, USA), enabling the production of a chimeric protein bearing an N-terminal 6×His affinity tag (Figure S6). The resulting recombinant plasmid, 6×His-pET-YEZV-N-D1, was transformed into chemically competent *E.coli* BL-21(DE3) cells (Thermo Fisher Scientific, Waltham, MA, USA). The accuracy of the final construct was verified by full-length Sanger sequencing.

### 4.2. Production and Purification of Recombinant YEZV Nucleoprotein Domain 1

Transformed cells were grown in LB medium (AppliChem, Darmstadt, Germany) supplemented with kanamycin (100 μg/mL) at 37 °C until an optical density of OD_600_ ~1.0 was reached. Expression was induced by adding IPTG (Thermo Fisher Scientific, Waltham, MA, USA) to a final concentration of 1 mM, and incubation was continued at a reduced temperature (16 °C, 180 rpm) for 20–24 h. The biomass was harvested by centrifugation (4500× *g*, 15 min, 4 °C).

The cell pellet was resuspended in lysis buffer (20 mM Tris, 500 mM NaCl, 20 mM imidazole, pH 7.5) with the addition of DNase (Biolabmix, Novosibirsk, Russia) and MgCl2 (New England BioLab, Ipswich, MA, USA) and a cocktail of protease inhibitors (Trans Gene Biotech, Beijing, China), disrupted by sonication on ice, and centrifuged (13,000× *g*, 30 min, 4 °C). The clarified supernatant was filtered through a 0.22 μm membrane and loaded onto a HisPur Ni-NTA Resin (Thermo Fisher Scientific, Waltham, MA, USA) for IMAC (HBBio-Lab 100 Chromatography System; Hanbon Sci. & Tech., Huaian, China). After washing away unbound proteins, the chimeric YEZV N D1 protein was eluted by stepwise increase in imidazole concentration (Figure S7a). The pooled fractions were dialyzed against a proteolysis-compatible buffer. The identity and specificity of the purified chimeric YEZV N D1 were subsequently confirmed by Western blot analysis according to Section Western Blot Analysis (Figure S8).

Removal of the 6×His tag was performed using recombinant rhinovirus A28 3C protease (SRC VB “Vector” Rospotrebnadzor, Koltsovo, Russia) at an enzyme-to-substrate molar ratio of 1:8 (4 °C, 16 h). Following proteolysis, the mixture was reapplied to a Ni-NTA column under reverse-IMAC, allowing the target YEZV N D1 (lacking N-terminal tag) to be collected in the flow-through fraction (Figure S7b). Eluted YEZV N D1 was concentrated using centrifugal filter units (5 kDa MWCO; Jet BioFil, Guangzhou, China) and purified using SEC with Superdex^®^200 Increase 10/300 GL (GE Healthcare, Stockholm, Sweden) in a buffer containing 20 mM Tris, 150 mM NaCl, and pH 7.5. Protein purity and molecular weight were assessed by SDS-PAGE in a 12% polyacrylamide gel according to Laemmli (Figure S7c).

#### Western Blot Analysis

Proteins were transferred from the gel to a nitrocellulose membrane using a WIX-fastBLOT Fast Semi-dry Blot (Wix Technology, Beijing, China) in transfer buffer (20 mM Tris, 192 mM glycine, 10% (*v*/*v*) ethanol) at 0.6 A for 90 min. Following transfer, membranes were blocked with EveryBlot Blocking Buffer (Bio-Rad, Hercules, CA, USA) for 1 h at room temperature. Membranes were then incubated overnight at 4 °C with Anti-6X His tag^®^ antibody (Abcam, Cambridge, UK) diluted 1:5000 in blocking buffer. After five washes (5 min each) with TBST (20 mM Tris-HCl pH 7.5, 150 mM NaCl, 0.1% (*v*/*v*) Tween 20), membranes were incubated for 1 h at room temperature with horseradish peroxidase (HRP)-conjugated goat anti-mouse IgG (H+L) secondary antibody (Thermo Fisher Scientific, Waltham, MA, USA) diluted 1:100,000 in blocking solution. Chemiluminescent detection was performed using SuperSignal™ West Pico PLUS Chemiluminescent Substrate (Thermo Fisher Scientific, Waltham, MA, USA). Signals were captured using a Li-Cor C-Digit Western Blot Scanner (LI-COR Biosciences, Lincoln, NE, USA) and quantified with Image Studio 5.2 software (LI-COR Biosciences, Lincoln, NE, USA) (Figure S8).

### 4.3. Dynamic Light Scattering (DLS) Measurement

DLS data were collected using a BeNano 180 Zeta Pro (Bettersize, Dandong, China) with a light source wavelength of 671 nm, a fixed scattering angle of 173°, and a temperature of 23–25 °C. The HullRad server (http://52.14.70.9/Run_hullrad.html; accessed on 20 March 2024) [21] was used for calculating hydrodynamic properties of AlphaFold-predicted structure and the theoretical molecular weight of protein.

### 4.4. SEC-SAXS Data Collection and Analysis

SEC-SAXS measurements for YEZV N D1 were performed at the BL19U2 beamline of the Shanghai Synchrotron Radiation Facility (SSRF, Shanghai, China) [22]. The X-ray beam size on the stage was 0.33 mm (H) × 0.05 mm (V). A two-dimensional Pilatus3 2M detector (DECTRIS Ltd., Baden, Switzerland) was placed at a sample-to-detector distance of 2.7 m. The scattering vector magnitude range (s = (4π/λ)sinθ, where 2θ is the scattering angle and λ = 0.1033 nm is the wavelength) was 0.07–4.5 nm^−1^.

SEC-SAXS was carried out using a Superdex^®^200 Increase 10/300 GL column at 20 °C with a flow rate of 0.5 mL/min. Chromatography was performed on an Agilent 1260 Infinity HPLC system (Agilent Technologies, Santa Clara, CA, USA). The YEZV N D1 sample was loaded at a concentration of 10.34 mg/mL, with an injection volume of 150 μL.

Data collection was performed by continuous acquisition of SAXS frames from the moment of sample injection onto the SEC column until the return to baseline. Primary data processing was carried out using the ATSAS v.4.0.1 with CHROMIXS (EMBL, Dublin, Ireland; https://www.embl-hamburg.de/biosaxs/; accessed on 1 December 2025), employed for automated analysis of SEC-SAXS datasets [23,24]. Background frames were selected from regions preceding and following the elution peak, followed by frame-by-frame buffer subtraction from each frame within the protein peak region. Frames corresponding to the monodisperse peak apex were merged for subsequent analysis.

Guinier analysis, pair-distance distribution function P(r) calculation, and ab initio shape reconstruction were performed using PRIMUS, GNOM, and DAMMIF, respectively [25,26,27]. The radius of gyration (Rg) and forward scattering intensity I(0) were determined from Guinier analysis (valid for sRg < 1.3; sRg = 0.3–1.3). Molecular weight (MW) was estimated using a Bayesian approach.

Ab initio low-resolution shape reconstruction was performed by approximating the molecular envelope with a system of dummy atoms using DAMMIF [27]. The resulting ab initio models were averaged and filtered against the experimental scattering curve using DAMAVER [28]. Superposition of the low-resolution envelope onto the atomic model of YEZV N D1 generated by AlphaFold was carried out using the CIFSUP [29], with model alignment optimized according to the normalized spatial discrepancy criterion. The excluded volume was estimated from ab initio modeling results generated by DAMMIF. Theoretical scattering curves from AlphaFold-predicted YEZV N D1 model was calculated with CRYSOL and GASBOR [30]. The goodness of fit was assessed using the reduced, weighted χ^2^ statistic.

### 4.5. Structure Prediction and MD Simulation

Structural models were predicted using AlphaFold3 v3.0.1 via the official AlphaFold Server (Google DeepMind, London, UK; https://alphafoldserver.com, accessed on 1 December 2025) [31]. Structural inferences were based on per-residue confidence scores provided via the AlphaFold 3 pLDDT metric and pTM metric. The pLDDT quantitatively estimates deviations in Cα–Cα interatomic distances between the reference and predicted structural models, with values ranging from 0 to 100. The pTM was used to assess the global structural accuracy of the predicted models, independent of residue-level confidence metrics. For models involving potential protein complex formation, the ipTM was additionally calculated to evaluate the reliability of intermolecular interface predictions. Models were selected for downstream analysis based on high-confidence thresholds established for three-dimensional structure prediction. Each selected model was spatially aligned with previously identified homologous proteins using structural superposition. The quality of structural alignment was evaluated using two key metrics: RMSD of Cα atoms, which quantifies the average atomic displacement upon superposition, and TM-score, which provides a length-independent measure of topological similarity. Tertiary structure models of viral proteins were visualized using UCSF ChimeraX v1.15rc (University of California, San Francisco, CA, USA) [32]. Comparison of secondary structures was visualized using the ESPript v3.0 software (Institute of Protein Biology and Chemistry, Lyon, France; https://espript.ibcp.fr/; accessed on 1 December 2025) [33].

The simulation system was automatically sized to accommodate the viral proteins with a minimum distance of 10.0 Å between any protein atom and the edge of a rectangular periodic boundary box. The system was solvated with explicit OPC water molecules. Counterions were added using a Monte Carlo placement algorithm to neutralize the net protein charge and achieve a physiological ionic strength of 150 mM NaCl. All simulations were performed using the AMBER ff19SB force field (Amber package, University of California, San Francisco, CA, USA).

System equilibration was carried out in two stages. First, an NVT ensemble simulation was conducted at 303.15 K for 125 ps with a 1.0 fs time step. Temperature was controlled using Langevin dynamics with a collision frequency of 1.0 ps^−1^, and protein heavy atoms were restrained with a harmonic force constant of 1.0 kcal·mol^−1^·Å^−2^. All covalent bonds involving hydrogen atoms were constrained using the SHAKE algorithm, and a nonbonded cutoff distance of 9.0 Å was employed. The equilibrated system was subsequently subjected to a 10 ns conventional NPT simulation at 303.15 K and 1 bar using a 2.0 fs integration time step. Pressure was maintained using a Monte Carlo barostat, while temperature control was achieved with the Langevin thermostat. After the conventional MD pre-run, accelerated molecular dynamics simulations were performed in the NPT ensemble using the dual-boost mode, in which both dihedral and total potential energy terms were modified to enhance conformational sampling. Following equilibration, a 500 ns production trajectory was generated. Trajectory analysis was performed using CPPTRAJ (Amber package, University of California, San Francisco, CA, USA; https://ambermd.org, accessed on 1 December 2025) [34]. Key parameters evaluated included RMSD of Cα atoms relative to the initial structure, RMSF, and Rg.

MD simulations of the YEZV N D1-ssRNA(-) complex and the dimeric YEZV N-ssRNA(-) complex were additionally performed for 300 ns. All MD simulations were carried out using the Amber package (University of California, San Francisco, CA, USA). Clustering analysis of the trajectories was carried out for both systems using 10 clusters. For the YEZV N D1-ssRNA(-) complex, clustering was performed based on conserved residues of the monomer and three nucleotides involved in the RNA-binding site. For the YEZV N dimer-ssRNA(-) complex, clustering was performed using Cα atoms. Protein–RNA interactions were analyzed using VMD v2.0 (University of Illinois, Urbana-Champaign, IL, USA).

## 5. Conclusions

The recombinant YEZV N D1 and its first experimentally validated solution structure reported here provide a critical foundation for understanding the molecular architecture of this emerging pathogen. While the domain’s conformational stability is firmly supported by SEC-SAXS data, it is important to note that the proposed RNA-binding interface is primarily derived from in silico molecular dynamics simulations and will require future in vitro experimental validation. Nevertheless, this structurally characterized protein lays a robust groundwork for the development of standardized serological assays and structure-guided therapeutic design against YEZV and related orthonairoviruses.

## 6. Patents

Tsishevskaya A.A., Gladysheva A.V. Artificial gene encoding domain 1 of Yezo virus nucleoprotein, recombinant plasmid DNA 6xHis-pET-Yezo_virus_d1_N-dop and recombinant producer strain *E.coli* BL-21/pET200_Yezo_virus_d1_N-dop, containing specified plasmid DNA, which ensures synthesis of chimeric protein Yezo_virus_d1_N-dop, used in production of recombinant domain 1 of Yezo virus nucleoprotein for structural and diagnostic studies. RU 2851093 C2, 18 November 2025.

## Data Availability

The SEC-SAXS dataset supporting this study has been deposited in the Zenodo repository and is publicly available at: https://doi.org/10.5281/zenodo.20606933. Further inquiries can be directed to the corresponding author.

## Supplementary Materials

The following supporting information can be downloaded at: https://www.mdpi.com/article/10.3390/ijms27125492/s1.

## Abbreviations

The following abbreviations are used in this manuscript:

YEZV	Yezo virus
N	Nucleoprotein
D1	Domain 1
YEZV N D1	Yezo virus nucleoprotein domain 1
SEC-SAXS	Small-angle X-ray scattering
ssRNA(-)	Single-stranded RNA of negative polarity
S	Small segment of the viral genome
M	Medium segment of the viral genome
L	Large segment of the viral genome
GPC	Glycoprotein precursor
RdRp	RNA-dependent RNA polymerase
RNP	Ribonucleoprotein
CCHFV	Crimean-Congo hemorrhagic fever virus
BJNV	Beiji nairovirus
SGLV	Songling virus
MD	Molecular dynamic
SEC	Size exclusion chromatography
DLS	Dynamic light scattering
Rh	Hydrodynamic radius
pLDDT	Local Distance Difference Test
RMSD	Root-mean-square deviation
Rg	Radius of gyration
ipTM	Predicted interface TM-score
TM-score	Template Modeling score

## References

[1] Yamaguchi H., Mizuma K., Watari K., Ohari Y., Mitsuhashi K., Tamiya K., Kawaguchi N., Orba Y., Saijo M., Matsuno K. (2026). Epidemiological, Clinical, and Virological Characterizations of Yezo Virus Infections, an Emerging Tick-borne Orthonairovirus Disease in Japan. J. Infect. Dis..

[2] Apanasevich M., Dubovitskiy N., Derko A., Khozyainova A., Tarasov A., Kokhanenko A., Artemov G., Denisov E., Shestopalov A., Sharshov K. (2025). Genomic Characterization of a Novel Yezo Virus Revealed in Ixodes pavlovskyi Tick Virome in Western Siberia. Viruses.

[3] Kartashov M., Svirin K., Zheleznova A., Yanshin A., Radchenko N., Kurushina V., Tregubchak T., Maksimenko L., Sivay M., Ternovoi V. (2025). First Report of the Yezo Virus Isolates Detection in Russia. Viruses.

[4] Leijon M., Guevara L., Averhed G., Engman M.N., Banihashem F., Cervin L., Bäcklin B.-M., Anderson N.E., Zohari S., Neimanis A. (2026). A Novel Orthonairovirus Detected in Grey Seal (*Halichoerus grypus*). One Health.

[5] Wang Y., Dutta S., Karlberg H., Devignot S., Weber F., Hao Q., Tan Y.J., Mirazimi A., Kotaka M. (2012). Structure of Crimean-Congo Hemorrhagic Fever Virus Nucleoprotein: Superhelical Homo-Oligomers and the Role of Caspase-3 Cleavage. J. Virol..

[6] Jeeva S., Mir S., Velasquez A., Ragan J., Leka A., Wu S., Sevarany A.T., Royster A.D., Almeida N.A., Chan F. (2019). Functional Interaction between Nairovirus N and HSP70/Related CCHFV Nucleocapsid Protein Research. J. Biol. Chem..

[7] Yanshin A.O., Ivkina D.I., Tuyrin V.Y., Osinkina I.A., Tishin A.E., Olkin S.E., Ukladov E.O., Radchenko N.S., Arkhipov S.G., Ryzhykau Y.L. (2025). Structural Features of Nucleoproteins from the Recently Discovered Orthonairovirus songlingense and Norwavirus beijiense. Int. J. Mol. Sci..

[8] Rahman S., Chiou C.-C., Almutairi M.M., Ajmal A., Batool S., Javed B., Tanaka T., Chen C.-C., Alouffi A., Ali A. (2024). Targeting Yezo Virus Structural Proteins for Multi-Epitope Vaccine Design Using Immunoinformatics Approach. Viruses.

[9] Hassan A., Muhammad S., Asad Ali S., Syed Ainul A., Faisal A., Muhammad A., Sajjad A., Asia N., Hanbal A., Inam H. (2025). Molecular Modeling to Design a Multiepitope Vaccine against Emerging Tick-Borne Yezo Virus and Its Validation Through Biophysics Techniques. Silico Pharmacol..

[10] Kodama F., Yamaguchi H., Park E., Tatemoto K., Sashika M., Nakao R., Terauchi Y., Mizuma K., Orba Y., Kariwa H. (2021). A novel nairovirus associated with acute febrile illness in Hokkaido, Japan. Nat. Commun..

[11] Kuhn J.H., Alkhovsky S.V., Avšič-Županc T., Bergeron É., Burt F., Ergünay K., Garrison A.R., Marklewitz M., Mirazimi A., Papa A. (2024). ICTV Virus Taxonomy Profile: Nairoviridae 2024. J. Gen. Virol..

[12] Ohta K., Saka N., Nishio M. (2024). Identification of critical residues for RNA binding of nairovirus nucleoprotein. J. Virol..

[13] Wang W., Liu X., Wang X., Dong H., Ma C., Wang J., Liu B., Mao Y., Wang Y., Li T. (2015). Structural and Functional Diversity of Nairovirus-Encoded Nucleoproteins. J. Virol..

[14] Surtees R., Dowall S.D., Shaw A., Armstrong S., Hewson R., Carroll M.W., Mankouri J., Edwards T.A., Hiscox J.A., Barr J.N. (2016). Heat Shock Protein 70 Family Members Interact with Crimean-Congo Hemorrhagic Fever Virus and Hazara Virus Nucleocapsid Proteins and Perform a Functional Role in the Nairovirus Replication Cycle. J. Virol..

[15] Ohta K., Saka N., Fukasawa M., Nishio M. (2023). Hazara Orthonairovirus Nucleoprotein Facilitates Viral Cell-to-Cell Spread by Modulating Tight Junction Protein, Claudin-1. Front. Microbiol..

[16] Goedhals D., Paweska J.T., Burt F.J. (2014). Long-Lived CD8+ T Cell Responses Following Crimean-Congo Haemorrhagic Fever Virus Infection. PLoS Negl. Trop. Dis..

[17] Leventhal S.S., Bisom T., Clift D., Rao D., Meade-White K., Shaia C., Murray J., Mihalakakos E.A., Hinkley T., Reynolds S.J. (2024). Antibodies targeting the Crimean-Congo Hemorrhagic Fever Virus nucleoprotein protect via TRIM21. Nat. Commun..

[18] Pirincal A., Keskin S., Doymaz M.Z. (2026). Amino Terminal Region of Crimean-Congo Hemorrhagic Fever Virus (CCHFV) Nucleocapsid (NP) Protein Contains Dominant Epitopes Recognised by Cellular Immunity. Immunology.

[19] Pirincal A., Doymaz M.Z. (2024). The Role of Nucleocapsid Protein (NP) in the Immunology of Crimean–Congo Hemorrhagic Fever Virus (CCHFV). Viruses.

[20] Álvarez-Rodríguez B., Tiede C., Hoste A.C.R., Surtees R.A., Trinh C.H., Slack G.S., Chamberlain J., Hewson R., Fresco A., Sastre P. (2020). Characterization and Applications of a Crimean-Congo Hemorrhagic Fever Virus Nucleoprotein-Specific Affimer: Inhibitory Effects in Viral Replication and Development of Colorimetric Diagnostic Tests. PLoS Negl. Trop. Dis..

[21] Fleming P.J., Fleming K.G. (2018). HullRad: Fast Calculations of Folded and Disordered Protein and Nucleic Acid Hydrodynamic Properties. Biophys. J..

[22] Li Y.-W., Liu G.-F., Wu H.-J., Zhou P., Hong C.-X., Li N., Bian F.-G. (2020). BL19U2: Small-Angle X-Ray Scattering Beamline for Biological Macromolecules in Solution at SSRF. Nucl. Sci. Tech..

[23] Petoukhov M.V., Franke D., Shkumatov A.V., Tria G., Kikhney A.G., Gajda M., Gorba C., Mertens H.D.T., Konarev P.V., Svergun D.I. (2012). New Developments in the ATSAS Program Package for Small-Angle Scattering Data Analysis. J. Appl. Crystallogr..

[24] Panjkovich A., Svergun D.I. (2018). CHROMIXS: Automatic and Interactive Analysis of Chromatography-Coupled Small-Angle X-Ray Scattering Data. Bioinformatics.

[25] Konarev P.V., Volkov V.V., Sokolova A.V., Koch M.H.J., Svergun D.I. (2003). PRIMUS: A Windows PC-Based System for Small-Angle Scattering Data Analysis. J. Appl. Crystallogr..

[26] Svergun D.I. (1992). Determination of the Regularization Parameter in Indirect-Transform Methods Using Perceptual Criteria. J. Appl. Crystallogr..

[27] Svergun D. (1999). Restoring Low Resolution Structure of Biological Macromolecules from Solution Scattering Using Simulated Annealing. Biophys. J..

[28] Volkov V.V., Svergun D.I. (2003). Uniqueness of ab initio Shape Determination in Small-Angle Scattering. J. Appl. Crystallogr..

[29] Kozin M.B., Svergun D.I. (2001). Automated Matching of High- and Low-Resolution Structural Models. J. Appl. Crystallogr..

[30] Svergun D.I., Barberato C., Koch M.H.J. (1995). CRYSOL—A Program to Evaluate X-ray Solution Scattering of Biological Macromolecules from Atomic Coordinates. J. Appl. Crystallogr..

[31] Abramson J., Adler J., Dunger J., Evans R., Green T., Pritzel A., Ronneberger O., Willmore L., Ballard A.J., Bambrick J. (2024). Accurate Structure Prediction of Biomolecular Interactions with AlphaFold 3. Nature.

[32] Pettersen E.F., Goddard T.D., Huang C.C., Meng E.C., Couch G.S., Croll T.I., Morris J.H., Ferrin T.E. (2020). UCSF ChimeraX: Structure Visualization for Researchers, Educators, and Developers. Protein Sci..

[33] Robert X., Gouet P. (2014). Deciphering key features in protein structures with the new ENDscript server. Nucleic Acids Res..

[34] Case D.A., Aktulga H.M., Belfon K., Cerutti D.S., Cisneros G.A., Cruzeiro V.W.D., Forouzesh N., Giese T.J., Götz A.W., Gohlke H. (2023). AmberTools. J. Chem. Inf. Model..

