# Supplementary material for: Solution Structure of Nucleoprotein Domain 1 from the Emerging Yezo Virus
Source: Int J Mol Sci. 2026 Jun 18;27(12):5492. doi: 10.3390/ijms27125492 (PMC13300033; doi:10.3390/ijms27125492)
Supplement: Supplementary file 1 [file ijms-27-05492-s001.zip › ijms-4374516-supplementary.pdf]

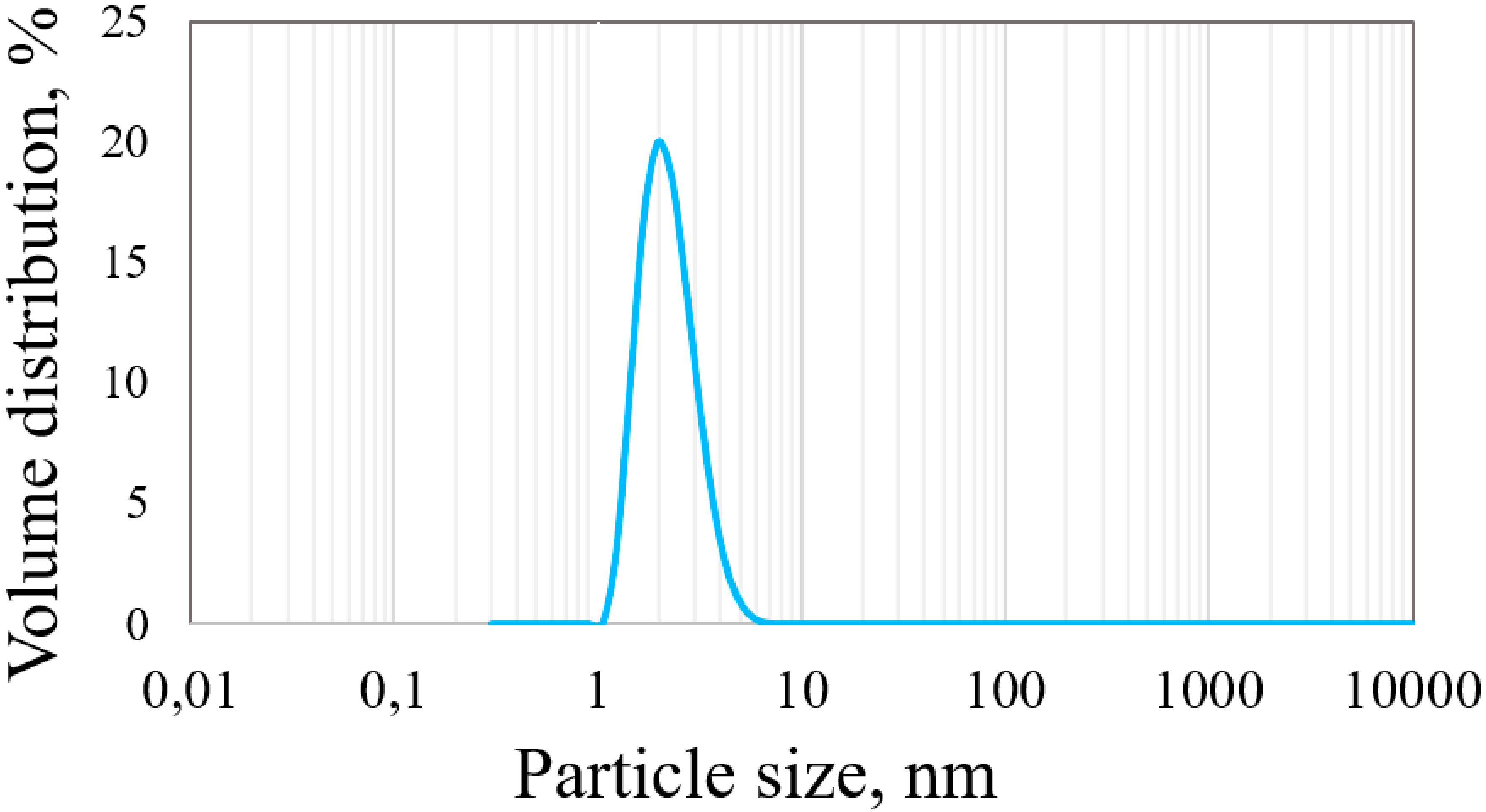

Figure S1. Dynamic light scattering analysis of the recombinant YEZV N D1 showing a monodisperse population with a hydrodynamic radius of 1.86 ± 0.4 nm.

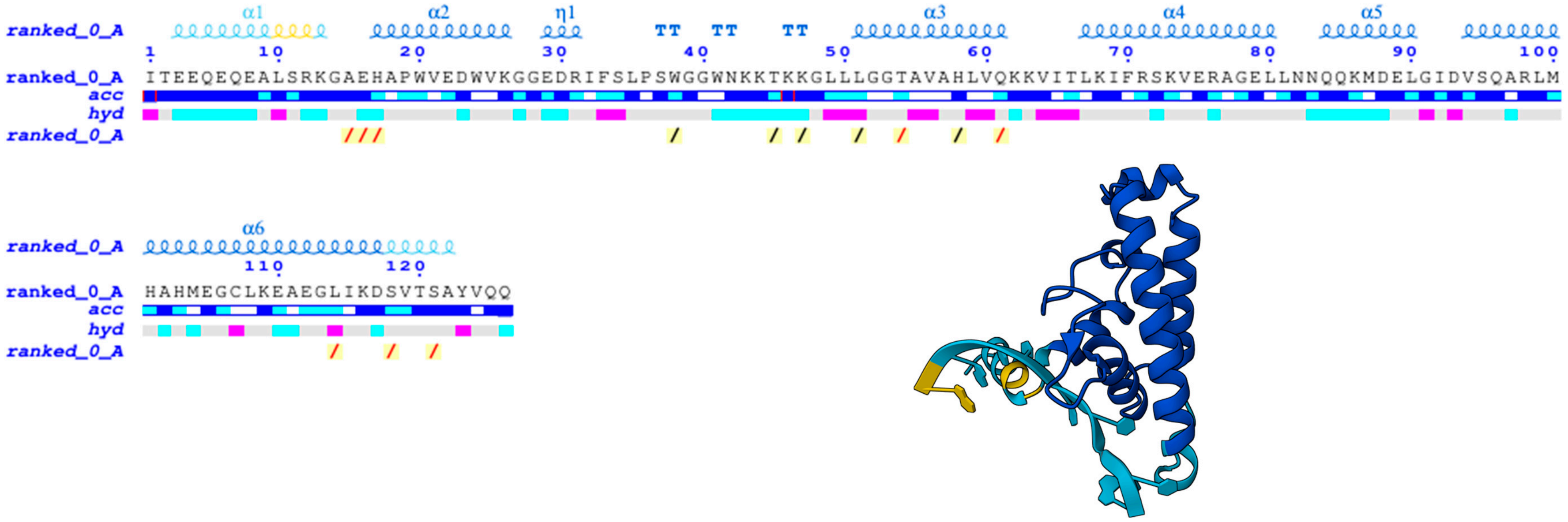

Figure S2. YEZV N D1-ssRNA(-) contacts. Secondary and tertiary structures are color-coded according to pLDDT, with blue indicating very high confidence and yellow indicating low confidence. The relative accessibility (labelled 'acc') calculated by DSSP for each residue is shown with a colored bar below the sequences block: white is buried, cyan is intermediate, blue is accessible. The hydropathy (labelled 'hyd') calculated from the query sequence using the Kyte&Doolittle algorithm is shown by a second coloured bar below the accessibility: pink is hydrophobic, grey is intermediate and cyan is hydrophilic. A "/" symbol indicate that the amino acid residue in question has a contact with RNA. A red "/" symbol indicates a contact < 3.2 Å. A black "/" symbol indicates a contact between 3.2 Å and 3.7 Å.

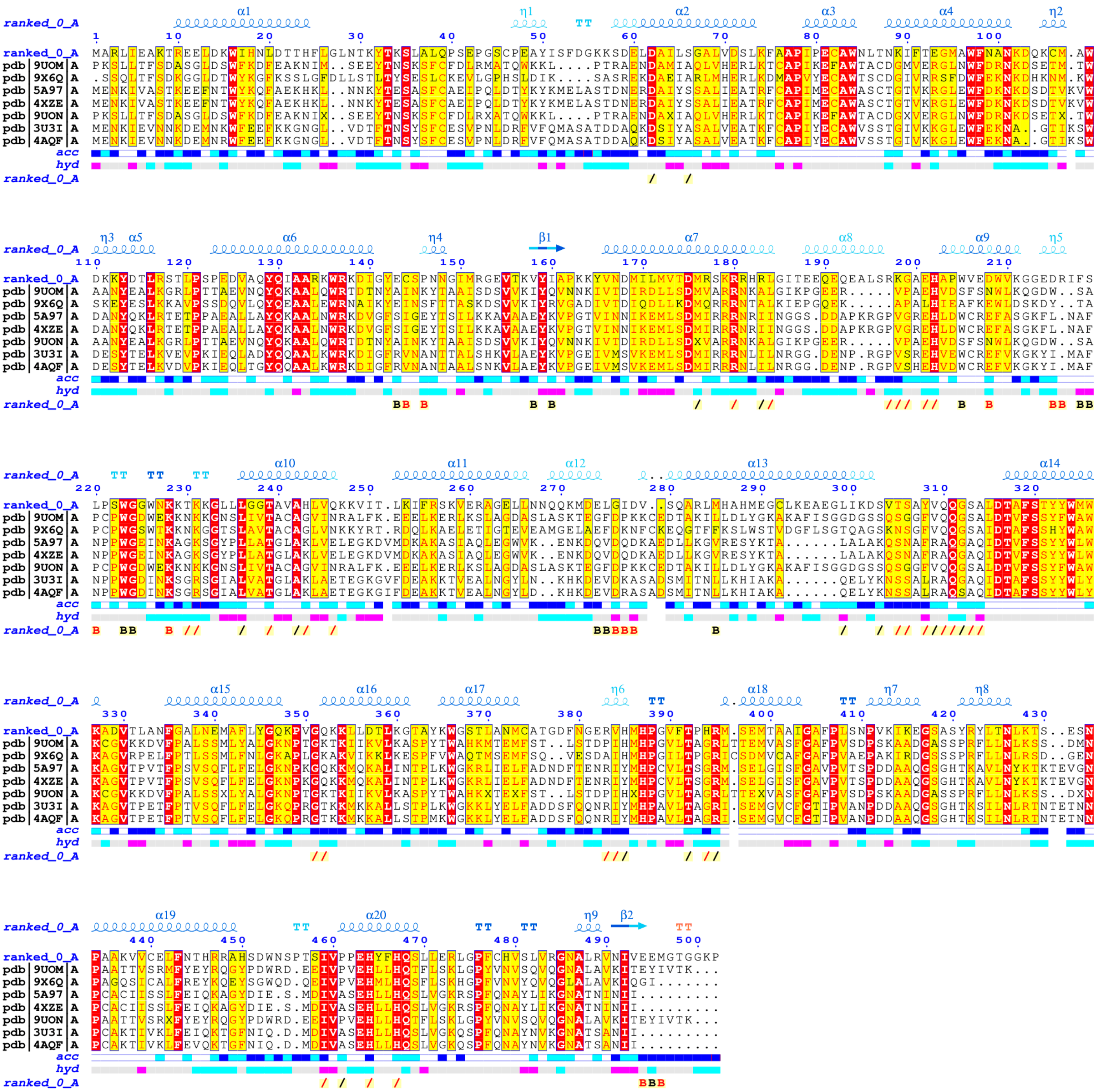

Figure S3. Sequence and structural annotation of the YEZV N dimer in complex with ssRNA(-) and homologous viral structures from PDB. Secondary and tertiary structures are color-coded according to pLDDT, with blue indicating very high confidence and yellow indicating low confidence. The relative accessibility (labelled 'acc') calculated by DSSP for each residue is shown with a colored bar below the sequences block: white is buried, cyan is intermediate, blue is accessible. The hydropathy (labelled 'hyd') calculated from the query sequence using the Kyte&Doolittle algorithm is shown by a second coloured bar below the accessibility: pink is hydrophobic, grey is intermediate and cyan is hydrophilic. A "/" symbol indicate that the amino acid residue in question has a contact with RNA. A red "/" symbol indicates a contact < 3.2 Å. A black "/" symbol indicates a contact between 3.2 Å and 3.7 Å.

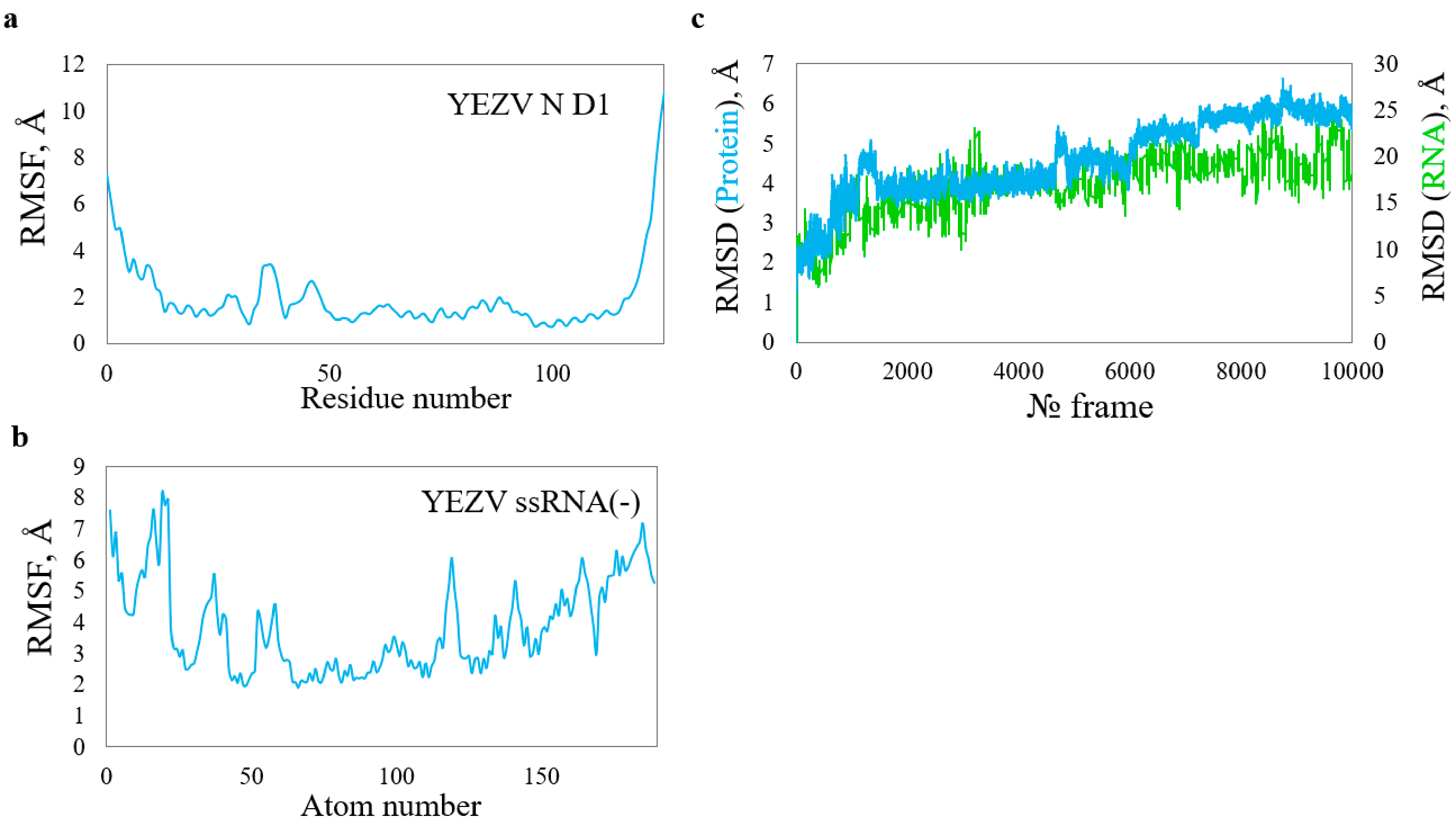

Figure S4. Structural clustering and RMSD analysis of the YEZV N D1-ssRNA(-) complex during molecular dynamics simulations. **(a)** RMSF profile of the protein calculated using Cα atoms. **(b)** RMSF profile of the RNA component. **(c)** RMSD trajectories of the protein (blue) and RNA (green) throughout the simulation.

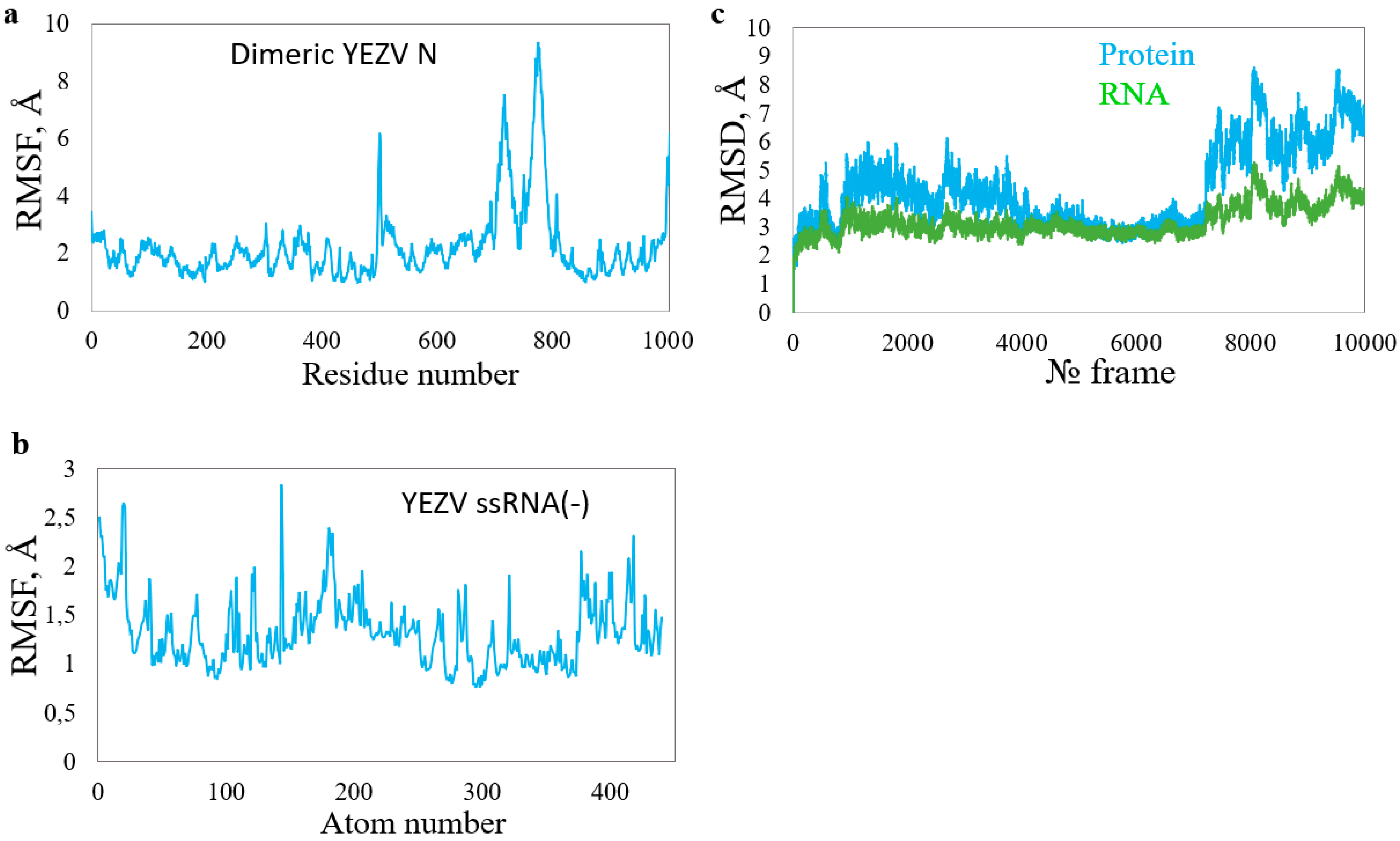

Figure S5. Structural clustering and RMSD analysis of the dimeric YEZV N-ssRNA(-) complex during molecular dynamics simulations. **(a)** RMSF profile of the protein calculated using Cα atoms. **(b)** RMSF profile of the RNA component. **(c)** RMSD trajectories of the protein (blue) and RNA (green) throughout the simulation.

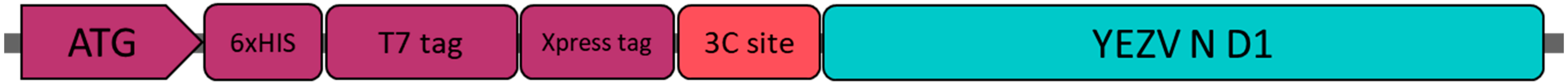

Figure S6. Schematic representation of the expression 6×His-pET-YEZV-N-D1 construct. The cassette encodes an ATG start codon, an N-terminal 6×His affinity tag (purple), a human rhinovirus 3C (HRV 3C) protease cleavage site (red), and the YEZV N D1 coding sequence (cyan).

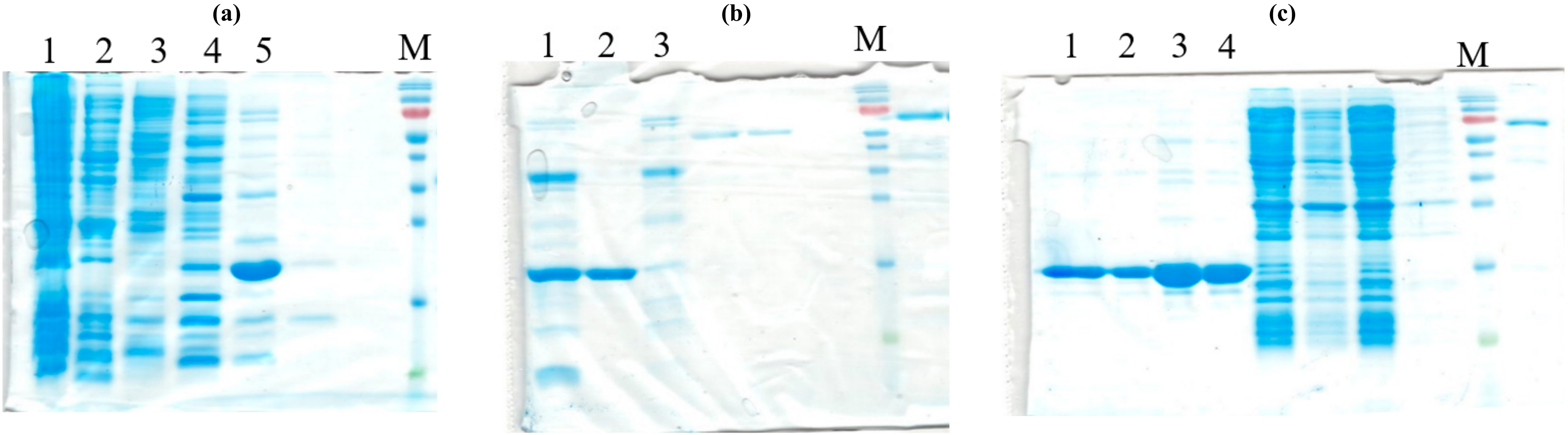

Figure S7. SDS-PAGE analysis of the recombinant YEZV N D1. **(a)**. SDS-PAGE analysis of IMAC purification step. Lanes: 1 - total cell lysate; 2 - column flow-through; 3-5 - step-gradient elution fractions with 50, 150, and 500 mM imidazole, respectively. **(b)**. SDS-PAGE analysis of the reverse IMAC (flow-through mode) following proteolytic cleavage. Lanes: 1 - proteolyzed sample; 2 - flow-through fraction containing the target tag-free YEZV N D1 protein; 3 - 500 mM imidazole elution fraction retaining the cleaved 6×His tag and HRV 3C protease. **(c)**. SDS-PAGE analysis of concentrated YEZV N D1 protein fractions after SEC (lanes 1-4).

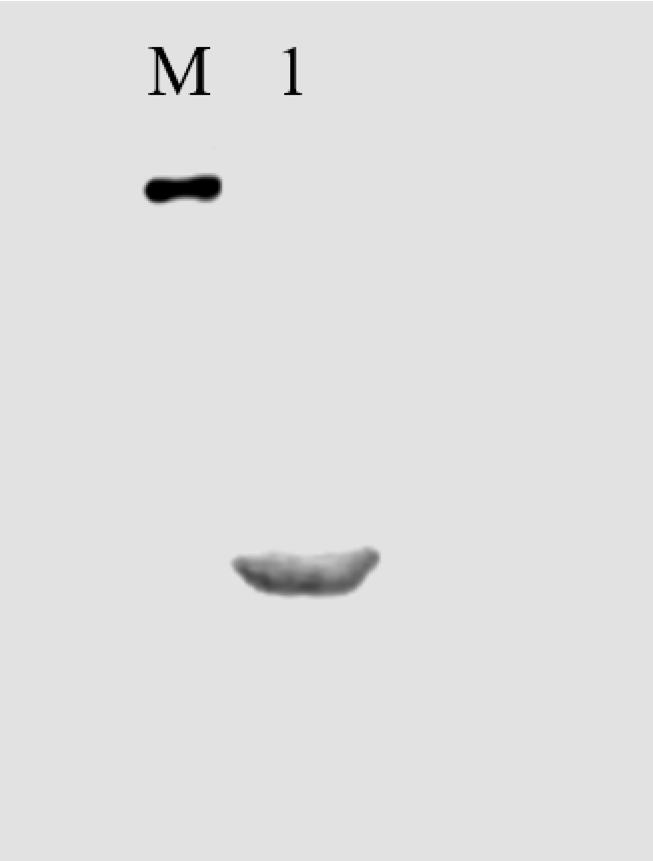

Figure S8. Western blot analysis of the chimeric YEZV N D1 sample probed with anti-6×His antibodies (lane 1).
